# Diesel exhaust particles increase IL-1β-induced human β-defensin expression via NF-κB-mediated pathway in human lung epithelial cells

**DOI:** 10.1186/1743-8977-3-9

**Published:** 2006-05-25

**Authors:** Hae Yun Nam, Eun-Kyung Ahn, Hyung Jung Kim, Young Lim, Chun Beoun Lee, Kyo Young Lee, Val Vallyathan

**Affiliations:** 1 Catholic Neuroscience Center, The Catholic University of Korea, Seoul 137–701, Korea; 2 Department of Occupational & Environmental Medicine, St. Mary's Hospital, The Catholic University of Korea, Seoul 150–713, Korea; 3 Department of Internal Medicine, Yonsei University College of Medicine, Seoul 135–270, Korea; 4 Environmental Parts R & D Center, Korea Automotive Technology Instititute, Cheonan, 330–912, Korea; 5 Department of Hospital Pathology, Kangnam St. Mary's Hospital, The Catholic University of Korea, Seoul 137–701, Korea; 6 Exposure Assessment Branch and Pathology and Physiology Research Branch, Health Effects Laboratory Division, National Institute for Occupational Safety and Health, Morgantown, WV, USA

## Abstract

**Background:**

Human β-defensin (hBD)-2, antimicrobial peptide primarily induced in epithelial cells, is a key factor in the innate immune response of the respiratory tract. Several studies showed increased defensin levels in both inflammatory lung diseases, such as cystic fibrosis, diffuse panbronchiolitis, idiopathic pulmonary fibrosis and acute respiratory distress syndrome, and infectious diseases. Recently, epidemiologic studies have demonstrated acute and serious adverse effects of particulate air pollution on respiratory health, especially in people with pre-existing inflammatory lung disease. To elucidate the effect of diesel exhaust particles (DEP) on pulmonary innate immune response, we investigated the hBD-2 and interleukin-8 (IL-8) expression to DEP exposure in interleukin-1 beta (IL-1β)-stimulated A549 cells.

**Results:**

IL-1β markedly up-regulated the hBD-2 promoter activity, and the subsequent DEP exposure increased dose-dependently the expression of hBD-2 and inflammatory cytokine IL-8 at the transcriptional level. In addition, DEP further induced the NF-κB activation in IL-1β-stimulated A549 cells more rapidly than in unstimulated control cells, which was showed by nuclear translocation of p65 NF-κB and degradation of IκB-α. The experiment using two NF-κB inhibitors, PDTC and MG132, confirmed that this increase of hBD-2 expression following DEP exposure was regulated through NF-κB-mediated pathway.

**Conclusion:**

These results demonstrated that DEP exposure increases the expression of antimicrobial peptide and inflammatory cytokine at the transcriptional level in IL-1β-primed A549 epithelial cells and suggested that the increase is mediated at least partially through NF-κB activation. Therefore, DEP exposure may contribute to enhance the airway-responsiveness especially on the patients suffering from chronic respiratory disease.

## Background

Diesel exhaust particles (DEP), which are generated by heavy-duty diesel engines, are major constituents of the atmospheric respiratory particles of less than 2.5 μm (PM2.5) in industrialized urban areas. With diameters < 2.5 μm, these particles can remain airborne for long periods of time and get deposited in great numbers deeply in the lungs. To date, many studies have demonstrated that DEP exposure is linked to the incidence of pulmonary inflammation and allergic airway disease, particularly asthma, [1-4] and increased susceptibility to bacterial infection [5-7].

Some interesting epidemiologic data have shown that the health effects of DEP are easily observed in people with pre-existing inflammatory lung diseases such as bronchitis, asthma, chronic obstructive pulmonary disease, pneumonia and compromised immune system, and age over 65 year old [8,9]. Recently studies have been reported that DEP may cause a change in innate and T cell-mediated immune responses [7] and be a potent adjuvant for the development of Th2 response characteristic of allergy and asthma in animal experiments [10,11]. Another study showed that lung macrophages activated by LPS might promote further inflammation by an enhanced cytokine response to inhaled air particles [12,13]. This evidence indicates that DEP exposure influences lung susceptibility in the inflammatory milieu of environment lung diseases.

Host defense against infection involves a multitude of factors and cells that together form the elements of innate and acquired immunity [14,15]. Especially, innate immune system, the first line of host defense, consists of a range of pre-existing, rapidly mobilized host defenses. Pulmonary epithelial cells that are a primary interface in direct contact with the ambient environment are a crucial site for this innate immune response. These cells generate various immune effectors such as cytokines, chemokines and antimicrobial peptides in response to inflammatory stimuli, and regulate the activation and recruitment of phagocytes, including neutrophils and macrophages, and immune cells with T cells and dendritic cells.

Antimicrobial peptides are key effector molecules in the innate immune system of the lung by virtue of their broad-spectrum microbicidal activity. Based on their structural characteristics, human defensins are divided into two subfamilies: α-defensin and β-defensin. Human α-defensins are mainly stored in the granules of phagocytes and Paneth cells, whereas human β-defensins (hBDs) are largely expressed in various epithelial tissues, including lung and skin [15-17]. The first hBD (hBD-1) was isolated in 1995 from hemofiltrate, and was later shown to be present in epithelial cells in various organs, entirely in a constitutive manner. The second hBD (hBD-2), initially found in psoriatic skin, is detected in airway surface fluid from patients with infectious lung disease but not from normal volunteers [18-20]. The mRNA for hBD-2 is present in the human lung and up-regulated by chronic inflammation, bacterial LPS or a proinflammatory mediator IL-1β. Other studies [21,22] demonstrated that the expression of hBD-2 in epithelial cells is amplified by LPS-stimulated monocytic cells through the production of IL-1β and TNF-α.

Most studies on defensins have mainly focused on their direct antimicrobial activity. However, a wide variety of recent *in vitro *and *in vivo *studies have illustrated the capacity of defensins to modulate the immunological and inflammatory responses [20,23], suggesting that their activities in the innate immune system may be closely linked to the inflammatory process.

Considering the roles of defensins in the initial defence response, it is important to elucidate the molecular mechanisms regulating their expression in a condition susceptible to external stimulus. In the present study, we focused on the effect of DEP on the inducible expression of hBD-2 and an inflammatory cytokine IL-8 in IL-1β-primed A549 lung epithelial cells. The results showed that DEP up-regulated the expression of endogenous hBD-2 and IL-8 mRNA in A549 cells and that the increase of hBD-2 expression was mediated through activation of NF-κB. This study provides insights into the pathogenesis of inflammatory lung disease following DEP exposure.

## Results

### Activation of hBD-2 promoter in IL-1β-stimulated A549 epithelial cells

Lung epithelial cells have been reported to participate in innate host defense by expressing antimicrobial peptide hBD-2 in response to inflammatory stimuli such as LPS and proinflammatory cytokines [14,19,22]

**Figure 1:**
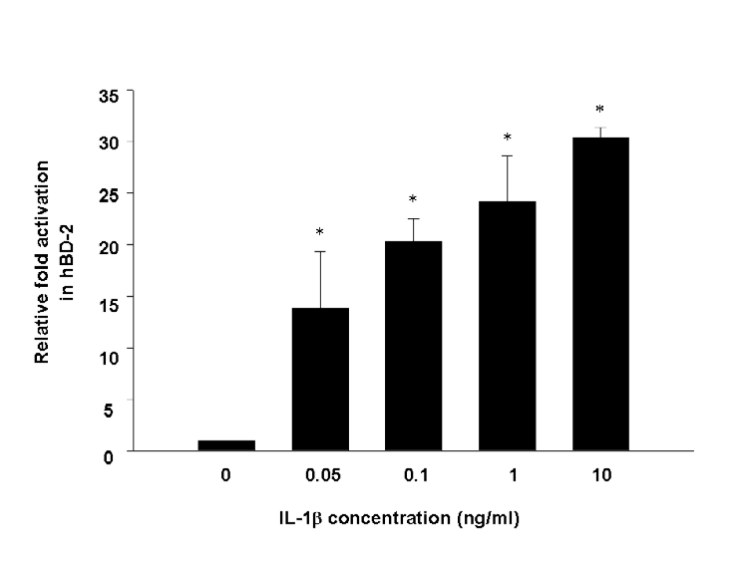
Induction of hBD-2 promoter activity by proinflammatory cytokine IL-1β in A549 cells. A549 cells were transfected with the luciferase plasmid containing hBD-2 promoter for 20 h and stimulated with the indicated concentrations of IL-1 β for 6 h. The cells were harvested and assayed for luciferase activity as described in Methods. Luciferase activities were expressed as a fold-activation relative to the un-stimulated control. Values are mean ± SD of five independent experiments. *, *P *< 0.0001.

We tried to evaluate the ability of A549 alveolar type II epithelial cells to induce hBD-2 expression following IL-1β stimulation and/or DEP exposure. As shown in Figure 1, hBD-2 promoter activity was increased dose-dependently after treatment with IL-1β (50–10,000 pg/ml) for 6 h. However, treatment of DEP (1–100 μg/ml) or LPS (up to 100 μg/ml) did not significantly induce activation of hBD-2 promoter (data not shown), which was consistent with the published results in the same cell line [22,24]. These results suggest that A549 epithelial cells may be sensitive to proinflammatory cytokine IL-1β. In a subsequent experiment, we used IL-1β as an activator to induce hBD-2 expression.

### DEP increased IL-1β-induced hBD-2 expression in lung epithelial cells

**Figure 2:**
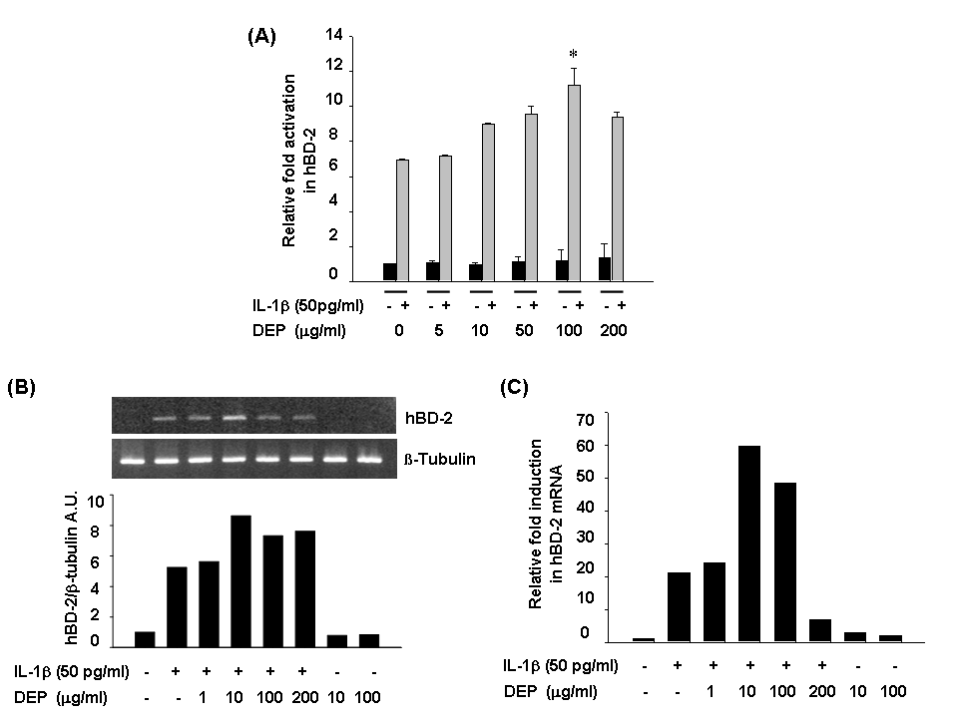
Effect of DEP on the expression of IL-1β-induced hBD-2 mRNA in A549 cells. (A) A549 cells were transfected with the luciferase plasmid containing hBD-2 promoter for 20 h. After pre-treatment with 50 pg/ml IL-1β for 1 h, the cells were exposed to 5–200 μg/ml of DEP for 24 h. The cells were harvested and assayed for luciferase activity as described in Methods. Luciferase activities are expressed as a fold-activation relative to the IL-1β only-treated control. Values are mean ± SD of five independent experiments. *, *P *< 0.005. Also, A549 cells were stimulated with 50 pg/ml IL-1β for 1 h and then treated with 5–200 μg/ml of DEP for 20 h. Total RNA was extracted and RT-PCR (B) and real-time quantification PCR (C) were performed using specific primers for hBD-2 and β-tubulin. The hBD-2 signals were normalized to β-tubulin and are presented in arbitrary units (A.U.).

To determine the effect of DEP on the IL-1β-induced hBD-2 expression, we pretreated A549 cells with IL-1β of 50 pg/ml, sufficient concentration to induce hBD-2 expression in our experiment and evaluated their response to DEP exposure. In the presence of IL-1β, DEP linearly increased the hBD-2 promoter activity over a concentration range of 5 to 100 μg/ml. Nevertheless, in its absence, DEP showed no change in the hBD-2 promoter activity (Figure 2(A)).

Next, we examined the endogenous level of hBD-2 mRNA following DEP exposure under the same culture condition. As shown in Figure 2(B), hBD-2 mRNA level was markedly increased, reached a peak level at 10 and 100 μg/ml DEP, and rapidly reduced at 200 μg/ml DEP.

Also, the degree of hBD-2 induction was quantitatively screened by real-time PCR. Consistent with the results of RT-PCR, DEP concentrations of 10 and 100 μg/ml increased the expression of hBD-2 mRNA by about 60- and 50-fold, respectively, in IL-1β-stimulated cells, whereas they did not at all in cells exposed to DEP alone (Figure 2(C)). Actually, a DEP dose of 200 μg/ml showed cell viability decreased to 62 and 85% in experiments using cytotoxicity assay and LDH release, respectively (data not shown), which may have contributed to a low expression level of hBD-2. These results indicate that DEP exposure increases IL-1β-induced hBD-2 expression at the transcriptional level in A549 cells.

### DEP increased IL-1β-induced IL-8 expression in lung epithelial cells

In the innate immune response, epithelial cells generate various immune effectors such as cytokines, chemokines, and nitric oxide, as well as antimicrobial peptides. Among cytokines, IL-8 is a pivotal inflammatory cytokine, which is involved in allergic inflammatory disorders such as asthma and has biological activities on eosinophils, the predominant cells in bronchial asthma, and neutrophils [25]. In this study, we examined the expression of IL-8 as an indicator to investigate the cellular inflammatory responses.

**Figure 3:**
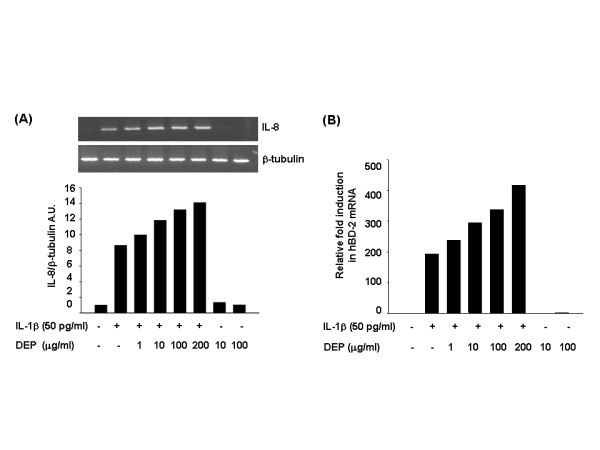
Effect of DEP on the expression of IL-1β-induced IL-8 mRNA in A549 cells. A549 cells were stimulated with 50 pg/ml IL-1β for 1 h and then treated with 1–200 μg/ml of DEP for 20 h. Total RNA was extracted as described in Methods. The mRNA levels of IL-8 and β-tubulin were analyzed by RT-PCR (A) and real-time quantification PCR (B). The IL-8 signals were normalized to β-tubulin and are presented in arbitrary units (A.U.).

As shown in Figure 3, DEP dose-dependently increased the expression level of endogenous IL-8 mRNA in IL-1β-stimulated cells, whereas DEP exposure alone showed almost no induction (Figure 3(A)). This was consistent with the results obtained in real-time PCR analysis (Figure 3(B)). However, compared to IL-1β-pretreatment control, DEP treatment did not induce any increase of IL-8 protein in culture media (data not shown). A similar discrepancy between mRNA expression and its protein secretion has been reported in other studies that detected the level of cytokines in the supernatant of cultured cells by ELISA method [23,26,27]. The authors postulated that these differences were caused by either the lack of the protease to cleave the immature form into the mature cytokine in the *in vitro *environment, or the intracellular storage or binding to the cell surface of cytokine. Taken together, these results indicated that DEP increase the expression of IL-8 at a transcriptional level in IL-1β-stimulated A549 cells, although the level of protein release was not correlated with that of mRNA expression.

### hBD-2 expression was mediated through NF-κB activation in IL-1β-stimulated A549 cells

IL-1β and TNF-α initiate NF-κB signaling and induce the transcription of several genes involved in innate immune response [22,27]. Based on these reports, we constructed the proximal promoter region of the hBD-2 gene containing three NF-κB binding motifs and addressed the involvement of NF-κB on hBD-2 regulation in IL-1β-stimulated A549 cells.

**Figure 4:**
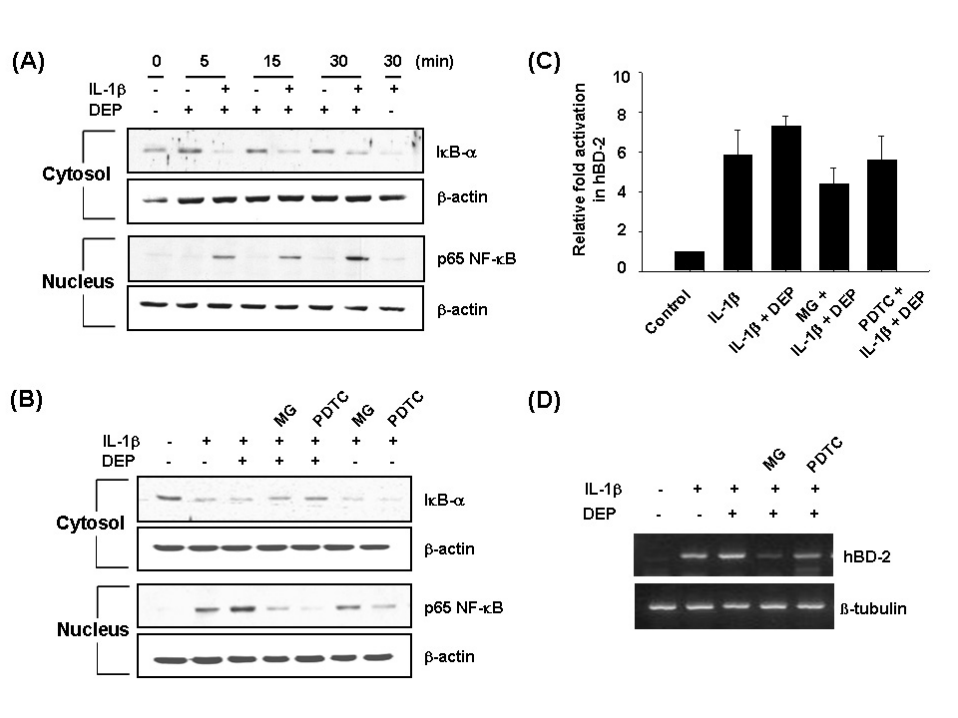
DEP regulate IL-1β-induced NF-κB activation in A549 cells. (A) A549 cells were stimulated with 50 pg/ml IL-1β for 1 h and treated with 100 μg/ml DEP for the indicated times. At each time point, the nuclear and cytosolic fractions were prepared and used for the analysis of p65 NF-κB, IκB-α and β-actin levels by Western blotting. (B) A549 cells were pretreated with MG132 (5 μM) or PDTC (100 μM) for 1 h and then stimulated with IL-1β (50 pg/ml) for 1 h followed by DEP treatment (100 μg/ml) for 5 min. The levels of p65 NF-κB, IκB-α and β-actin were analyzed by western blotting as mentioned above. (C) A549 cells were transfected with luciferase plasmid containing hBD-2 promoter for 20 h. The cells were pretreated with MG132 (5 μM) or PDTC (100 μM) for 1 h and then stimulated with IL-1β (50 pg/ml) for 1 h followed by DEP treatment (50 μg/ml) for 24 h. The cells were harvested and assayed for luciferase activity as described in Methods. Values are mean ± SD of four independent experiments. (D) A549 cells were pretreated with MG132 (5 μM) or PDTC (100 μM) for 1 h and then stimulated with IL-1β (50 pg/ml) for 1 h followed by DEP treatment (10 μg/ml) for 20 h. Total RNA was prepared and analyzed by RT-PCR using specific primers for hBD-2 and β-tubulin

In our study, the activation of NF-κB was assessed by the nuclear translocation of p65 NF-κB and the degradation of IκB-α, a NF-κB inhibitory protein. Treatment with 50 pg/ml IL-1β in A549 cells resulted in the marked nuclear translocation of p65 NF-κB and a concomitant degradation of IκB-α in the cytosol, which peaked at 5 min after IL-1β treatment and gradually recovered after 1 h (Figure 4(A)). In addition, subsequent DEP exposure synergically increased the IL-1β-induced NF-κB activation.

Using two NF-κB inhibitors, PDTC and MG132, we determined the involvement of NF-κB in IL-1β-induced hBD-2 expression. Pretreatment of NF-κB inhibitors effectively suppressed the IL-1β-induced nuclear translocation of p65 NF-κB and IκB-α degradation in A549 cells (Figure 4(B)). Furthermore, the IL-1β-induced hBD-2 mRNA expression and hBD-2 promoter activity were inhibited by pretreatment with inhibitors (Figure 4(C, D)). These results suggest that hBD-2 expression in lung epithelial cells is in part modulated by NF-κB transcription factor via the action of IL-1β.

## Discussion

Airway epithelial cells are constantly exposed to inhaled airborne pathogens and produce several effectors to protect the lung from them. Among these biological effectors, hBD-2 is an interesting antimicrobial peptide, which is able to modulate inflammatory responses and stimulate adaptive immunity in addition to the microbicidal activity in innate immunity [15,17,28]. In particular, it has been reported that under hyper-responsive inflammatory conditions such as asthma or viral infection, epithelial cells show a change of immune response to environmental particles exposure [29-31]. In this study, we examined the expression of hBD-2 and inflammatory cytokine IL-8 following DEP exposure in IL-1β-stimulated A549 lung epithelial cells and demonstrated the regulation of hBD-2 expression by NF-κB.

Consistent with previous reports that A549 cells were hyper-responsive to IL-1β [32,33], pretreatment of A549 cells with IL-1β markedly induced hBD-2 promoter activation at a low dose (50 pg/ml), while DEP alone showed no effect. Treatment of DEP in IL-1β-pretreated A549 cells increased the activity of hBD-2 promoter in a dose-dependent manner. In addition, we showed that DEP exposure increased hBD-2 and IL-8 expression at the transcriptional level in the same IL-1β-primed condition using RT-PCR and quantitative real-time PCR. These findings suggest that epithelial cells stimulated by IL-1β are sensitive to the exposure of subsequent environmental particles, DEP, which may result in increased expression of antimicrobial peptide and inflammatory cytokine at the transcriptional level.

In the lungs, while pulmonary epithelial cells rarely respond to bacteria or bacterial LPS, the cells readily respond to cytokines being triggered at a lower threshold [16,34,35]. Furthermore, recent studies have shown that epithelial cells modulate hBD transcription via pro-inflammatory cytokines (IL-1β and TNF-α) produced by LPS-stimulated, mononuclear phagocytes [21,22]. Previous studies [22,24,36] demonstrated that this hypo-responsiveness of epithelial cells to LPS was most likely due to lack of expression and membrane localization of toll-like receptor 4 (TLR4), which is required for recognition of bacterial component and signal transducer molecule to initiate the LPS signaling. Actually, we could not observe detectable levels of TLR4 in A549 cells by RT-PCR and western blotting assay, even after treatment with LPS (data not shown). Another study [24] suggested that epithelial cells that are in frequent contact with environmental stimuli (intestine and lung) are hypo-responsive to LPS at baseline and need a priming event to induce LPS responsiveness. In our study, we used IL-1β as a pro-inflammatory factor to induce responsiveness of A549 cells. The cells stimulated with a low dose of IL-1β (50 pg/ml) increased hBD-2 promoter activity up to 20 fold.

It has been also reported that transcriptional activation of hBD-2 is regulated in an NF-κB dependent manner [22,27]. The known genes regulated by NF-κB are pro-inflammatory cytokines, chemokines, antimicrobial peptides, inducible enzymes and adhesion molecules which are important effectors or mediators of innate and adaptive immune responses. We showed that IL-1β induced the activation of NF-κB via the degradation of IκB-α and the translocation of p65, a subunit of NF-κB, into the nucleus, and that subsequent DEP treatment increased the NF-κB activation time-dependently. In addition, we examined the involvement of NF-κB in hBD-2 transcription using two NF-κB inhibitors (MG132 and PDTC) with different pharmacological actions. Both inhibitors effectively reduced the hBD-2 expression induced by IL-1β alone and by the combination of IL-1β and DEP. These results indicate that hBD-2 is induced via activation of NF-κB in IL-1β-stimulated A549 cells and that DEP exposure up-regulates this mechanism of hBD-2 expression.

The previous studies demonstrated multiple regulatory pathways in β-defensin expression and regulation, which supports their pluripotential functional abilities. While initially perceived as being primarily antimicrobial in nature and contributing to the barrier protection of epithelial and mucosal surfaces, as suggested by the name 'defensin', it is now known that the β-defensin has an important role in not only the innate immune response, but also in immunoenhancing, inflammatory modulating and wound repairing capabilities. In a series of studies, elevated levels of α- and β-defensin, especially hBD-2, were observed in the plasma of bronchoalveolar lavage fluid (BALF) of patients with various inflammatory lung diseases [18,37,38]. Furthermore, recent *in vitro *studies have showed that the levels of defensin strongly correlate with those of the increased IL-8 from airway epithelial cells [23,39,40], which may contribute to some effect exerted by defensin on IL-8 synthesis and additional effects on lung chronic inflammation.

This is the first report to demonstrate the relationship between β-defensin and DEP-induced airway inflammation. Although our results did not show a direct relation between hBD-2 and IL-8 expression, we have suggested in this study that DEP exposure could synergistically induce increases of hBD-2 and IL-8 with chemotactic functions in IL-1β-primed lung epithelial cells, which may serve as crucial signal in mediating the initiation, mobilization and amplification of adaptive immune responses.

## Conclusion

This study showed that DEP, which constitute an important airborne pollutant in the urban environment, regulates the transcriptional expression of an antimicrobial peptide (hBD-2) and an inflammatory cytokine (IL-8) in IL-1β-stimulated A549 cells. From the finding, we have demonstrated the possibility of immunoenhancing in response to DEP exposure in lung epithelial cells. This suggests that the heightened inflammatory response might provide an insight to explain the adverse effect following particulate air pollution exposure in patients with pre-existing inflammatory lung disease. Finally, further investigation of intracellular response to DEP exposure will contribute to raising the understanding of the pulmonary innate immune system and the adverse health effects of DEP.

## Methods

### Reagents

The diesel particulate matter SRM 2975 was purchased from the National Institute of Standards and Technology (Gaithersburg, MD), and human recombinant IL-1β from R & D System (Minneapolis, MN). Antibodies against p65 NF-κB (sc-8008), IκB-α (sc-847), and β-tubulin (sc-5274) were obtained from Santa Cruz Biotechnology (Santa Cruz, CA), and the proteasome inhibitors, MG132 and pyrrolidine dithiocarbamate (PDTC), from Sigma-Aldrich (St. Louis, MO). MG132 and PDTC were dissolved in DMSO and further diluted in PBS to ensure a DMSO concentration not exceeding 0.1%.

### Cell cultures

The human lung alveolar type II epithelial cell line, A549 (CCL-185), was obtained from the American Type Culture Collection (Manassas, VA). A549 cells were maintained in RPMI-1640 medium (WelGENE, Daegu, South Korea) supplemented with 10% heat-inactivated fetal bovine serum (FBS), 2 mM glutamine, 100 U/ml penicillin and 0.1 mg/ml streptomycin (Invitrogen, Carlsbad, CA) in a humidified atmosphere of 95% air with 5% CO_2 _at 37°C. Typically, the cells were seeded and maintained in cell culture plates (Costar, Corning Inc., Corning, NY) for 24 h before stimulation with IL-1β and/or DEP. Negative controls were grown without the addition of IL-1β.

All cell culture exposures were performed in three separate experiments where each experiment was performed in triplicate.

### Preparation of DEP suspensions

The DEP suspension was prepared just prior to use. To induce particle disaggregation, stock solutions of particles were dispersed in PBS containing 0.05% Tween 80 (Sigma Chemical, St. Louis, MO) at a concentration of 10 mg/ml and then sonicated at output 5 and duty 30 of ultrasonic disrupter for 2 min under cooling conditions [41]. Different concentrations of particles were then diluted with RPMI-1640. Culture medium containing percentage of Tween 80 in the maximum concentration of DEP suspension was used as negative control in separate experiments.

### Plasmid construction

A 2.3-kbp fragment of human hBD-2 promoter (-2274 to +50) was amplified from genomic DNA by PCR using specific primers: forward 5'-GGC TCG AGG CTC AGA CAT CAG CAC CCA AA-3' (*Xho *I) and reverse 5'-AAA AGC TTC GAG AAG AGG AGA TAC AAG A-3' (*Hind *III). Both primers were designed based on GenBank Accession No, AF071216, and additional restriction sites are indicated by underlines. The synthesized fragments with the *Xho *I and *Hind *III sites were subcloned into the promoter-less firefly luciferase vector, pGL3-Basic (Promega, Madison, WI) to generate hBD-2/Luc plasmid.

### Transfection and luciferase assay

A549 cells (8 × 10^4 ^cells/well) were seeded into 12-well culture plates 1 day before transfection. Cells were transfected with 1.5 μg of firefly luciferase expression construct and 200 ng of *Renilla *luciferase expression vector pRL-TK (Promega) using transfectam reagent (Promega) according to the manufacturer's protocol. Following transfection, cells were cultured for 20 h, and then exposed to stimuli such as IL-1β and DEP.

After stimulation, cells were washed, harvested and lysed in 50 μl of passive lysis buffer. Firefly and *Renilla *luciferase activities were measured using a Dual-Luciferase reporter assay kit (Promega) and MicroLumat*Plus *LB96V luminometer (Berthold, Tokyo, Japan). Promoter activities are expressed as relative light units, normalized to *Renilla *luciferase activity.

### RT-PCR and real-time PCR analysis

Confluent A549 cells (8 × 10^5 ^cells/35-mm plate) were incubated with IL-1β or IL-1β and DEP for 20 h. Total RNA was then purified using an RNeasy mini kit (Qiagen, Valencia, CA) and treated with RNase-free DNase set (Qiagen) to remove contaminated DNA. RT-PCR was performed with 1.0 μg RNA using an AccuPower^® ^RT-PCR kit (Bioneer, Rockville, MD) according to the manufacturer's protocol in a thermal cycler (MJ Research, INC., Watertown, MA). The RT-PCR involved 38, 28 and 25 cycles for hBD-2, IL-8 and β-tubulin, respectively, of the following series: 30°C for 10 min, 42°C for 60 min, and 94°C for 5 min for RT; and 98°C for 10 s, 60°C for 10 s and 74°C for 30 s for PCR. To discriminate mRNA-derived PCR products from genomic DNA-derived products, the following intron-spanning PCR primers were used: hBD-2, forward 5'-CCA GCC ATC AGC CAT GAG GGT-3' and reverse 5'-GGA GCC ATC AGC CAT GAG GGT-3' (255 bp); IL-8, forward 5'-ATG ACT TCC AAG CTG GCG TG-3' and reverse 5'-TTA TGA ATT CTC AGC CCT CTT CAA AAA CTT CTC-3' (300 bp); and β-tubulin, forward 5'-GTT GGT CTG GAA TTC TGT GAG-3' and reverse 5'-AAG AAA TCC AAG CTG GAG TTC-3' (300 bp). All PCR products were analyzed on a 2% agarose gel.

To quantify the levels of hBD-2 and IL-8 mRNA expression, real-time PCR quantification was performed. In brief, 1 μg/ml of total RNA was reversed-transcribed to cDNA using an AccuPower^® ^RT PreMix (Bioneer). PCR reactions were performed in a total volume of 25 μl including 12.5 μl of 2 × IQ™ SYBR^® ^green supermix (Bio-Rad, Hercules, CA), 1 μl of cDNA and 1 μl of each primer (10 pm/μl). The amplification and detection were carried out in a Bio-Rad iCycler iQ system (Bio-Rad). The relative genomic copy number was calculated using the comparative threshold (Ct) method [42]. Briefly, the threshold cycle (*C*_T_) for each gene was determined using the thermocycler software and the average of three independent Cts/DNA was calculated. The copy number of the target gene normalized to an endogenous reference and relative to a calibrator is given by the formula 2^-ΔΔCT^. β-Tubulin was used as an endogenous reference.

### Western blotting

Cytosol/membrane protein fractions were obtained using NE-PER nuclear and cytoplasmic extraction reagents (PIERCE, Rockford, IL) according to the manufacturer's instruction. The protein concentration of extracts was determined with Bradford reagent (Bio-Rad) using bovine serum albumin as a standard. Equal amounts of extracts (40μg/lane) were resolved by 10% SDS-PAGE gel electrophoresis and transferred onto a nitrocellulose membrane. The membrane was then blocked with 5% non-fat dried milk in TTBS (Tris-buffered saline with 0.05% Tween 20) for 1 h and washed, and further incubated overnight with specific primary antibodies to p65 NF-κB, IκB-α and β-tubulin. The blots were washed four times with TTBS and incubated for 1 h with appropriate secondary antibodies coupled to horseradish peroxidase and developed in the ECL Western detection reagents (Amersham-Biosciences, Little Chalfont, Bucks., UK).

### Statistics

Data are expressed as means ± SD. For luciferase assay, differences between control and IL-1β and/or DEP-treated samples were compared by matched pair *t*-test. *P*-values < 0.05 were considered statistically significant.

## Competing interests

The author(s) declare that they have no competing interests.

## Authors' contributions

HYN had the initial idea of performing the studies, designed and coordinated the experimental work together with YL, who had edited the final version of the manuscript. EKA had carried out the cytotoxicity studies on DEP and supported the experiments. HJK and KYL are specialists of internal medicine and pathology respectively and have participated in the design of the study and the interpretation of the data. CBL supported collection of reference DEP emitted from automobiles engine and involved in the interpretation of the data. VV took the part of evaluation and review as the expert of pulmonary pathology, and commented on the final manuscript. All authors read, reviewed and approved final manuscripts.
